# The feasibility and radiological features of sacral alar iliac fixation in an adult population: a 3D imaging study

**DOI:** 10.7717/peerj.1587

**Published:** 2016-01-25

**Authors:** Ai-Min Wu, Yong-Long Chi, Wen-Fei Ni, Yi-Xing Huang

**Affiliations:** Department of Orthopaedic Surgery, Second Affiliated Hospital of Wenzhou Medical University, Zhejiang Spinal Research Center, Wenzhou, Zhejiang, China

**Keywords:** Sacral fracture, Radiological study, Sacral alar iliac screw, Adult population, 3D digital images, Sacral fracture

## Abstract

**Background:** Surgical treatments for adult spinal deformities often include pelvic fixation, and the feasibility of sacral-2 alar iliac (S2AI) screw fixation has been shown previously. However, sometimes S2AI screw fixation cannot be applied due to the presence of an osteolytic lesion or trauma or because the biomechanical properties of only an S2AI screw is insufficient. Therefore, we questioned the feasibility of using sacral AI screws in other segments and determined whether S3AI and S4AI screws have the potential to be used for sacral fractures. The aim of this study was to investigate the feasibility and radiological features of sacral AI fixation in S1–S4 in an adult population using 3D imaging techniques. **Methods:** Computed tomography (CT) scans were taken of 45 patients and were imported into Mimics (Version 10.01, Materialise, Belgium) software to reconstruct the 3D digital images. Next, a cylinder (radius of 3.5 mm) was drawn to imitate the screw trajectory of a S1–4 AI screw, and every imitated screw in each segment was adjusted to a maximum upward and downward angle to acquire the feasible region. The parameters of the S1–4AI screw trajectories were measured. **Results:** Sacral AI screws could be successfully imitated using 3D digital imaging. The S4AI screw trajectory could be obtained in 19 of 45 patient images (42.2%), while the feasibility rates of S1AI, S2AI, and S3AI screw fixation were 100%, 100%, and 91.1% (41/45), respectively. The feasible regions of S1AI, S2AI, and S3AI screw trajectories were wide enough, while the adjustable angle of S4AI screws was very small. **Conclusion:** It is feasible to place S1–2AI screws in the entire adult population and S3–4AI screws in some of the adult population. Furthermore, our study suggested that 3D digital images are suitable to study the feasibility of new screw fixation.

## Introduction

Surgical treatments for adult spinal deformity often include pelvic fixation, and the high rate of pseudarthrosis in distal fixation in spinal deformity is a challenge for spinal surgeons (Edwards et al., 2004; Edwards et al., 2003; Kim et al., 2006). A novel technique for sacral-2 alar iliac (S2AI) screw fixation has been introduced for distal fixation (O’Brien et al., 2009), and it has been reported that the S2AI screw can be inserted by an open or percutaneous approach (Martin, Witham & Kebaish, 2011; O’Brien et al., 2010). The percutaneous approach has the advantages of being minimally invasive, causing less blood loss, and having a lower rate of wound infection and quicker postoperative recovery times (Martin, Witham & Kebaish, 2011; Xu et al., 2013). In addition, the feasibility of S2AI screw fixation has been shown by previous studies (Kwan et al., 2012; O’Brien et al., 2010; Zhu et al., 2013).

However, sometimes the S2AI screw cannot be applied due to an osteolytic lesion or trauma (O’Brien et al., 2009) or because the biomechanical properties of only an S2AI screw are insufficient. Therefore, we questioned the feasibility of using sacral AI screws in other segments.

In this study, we used Mimics 3D imaging software (Version 10.01, Materialise, Belgium) to investigate the feasibility of S1–4AI screw fixation. This software has previously been shown to provide an accurate visual screw trajectory (Wu et al., 2013).

## Materials and Methods

This research was performed following the principles described in the Declaration of Helsinki and was approved by the Institutional Ethics Review Board of the Second Affiliated Hospital of Wenzhou Medical University (No. 2015–30). Written informed consent was obtained from all participants.

**Figure 1:**
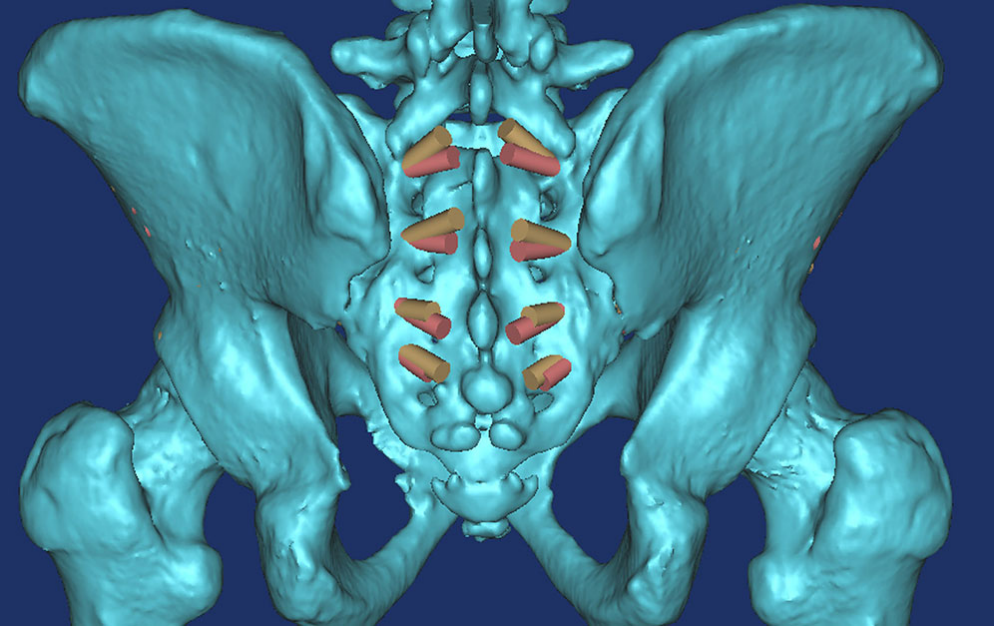
All of the S1AI-S4AI screw trajectories were simulated on 3D digital image; the ideal entry point was at the inner fovea of the transverse process of the sacrum.

Computed tomography (CT) scans (Dicom format) were taken of the pelvic regions of 45 patients and were imported into Mimics software (Version 10.01, Materialise, Belgium) for reconstructing the 3D digital images (Wu et al., 2014), which only takes about 10 minutes for one subject. The mean age of the patients was 53.6 ± 19.5 years old and ranged from 20 to 84 years old. Briefly, their CT scans in DICOM format were imported into Mimics software for three-dimensional (3D) reconstruction; the threshold value was set at “Bone (CT)” as “226-Max,” which is optimal for bone reconstruction. After the 3D digital images were calculated and reconstructed, a cylinder (radius of 3.5 mm) was drawn to imitate the screw trajectory of S1–4AI screws. The feasibilities of S1–4AI screw fixation were first observed by adjusting the angle and the ideal entry point. The ideal entry point was at the inner fovea of the transverse process of the sacrum (Fig. 1), from S1 to S4, and it was a line, not limited to a determined point. To point the screw trajectory away from the dorsal sacral foramina, we chose the screw entry point as the cross of the middle line between the upper and lower dorsal sacral foramina and the line of the inner fovea of the transverse process of the sacrum. After the screw entry point was determined, every imitated screw in each segment was adjusted to the maximum upward and downward angles, which means the screw trajectories were adjusted maximum upward or downward, but not penetrated out of the cortex of the pelvic bone.

**Figure 2:**
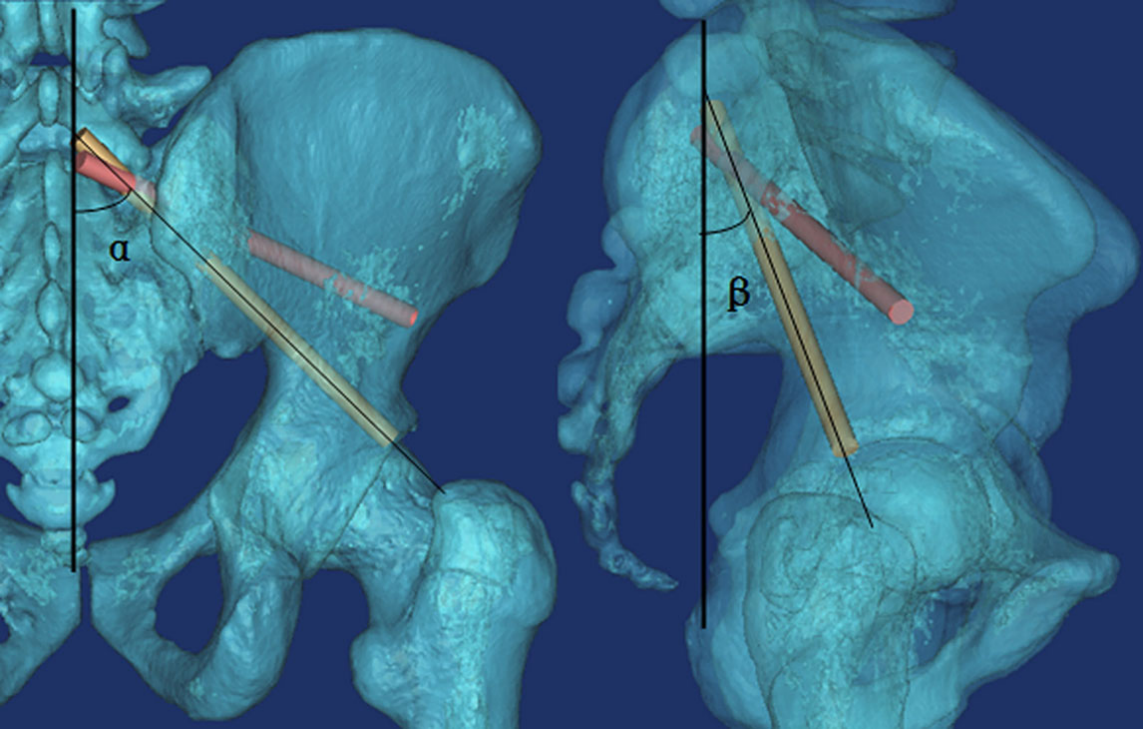
(A) The angle between the screw trajectory and the erect line from the anteroposterior view; (B) The angle between the screw trajectory and the erect line from the lateral view. (The yellow trajectory is the maximum downward screw; the red trajectory is the maximum upward screw).

**Figure 3:**
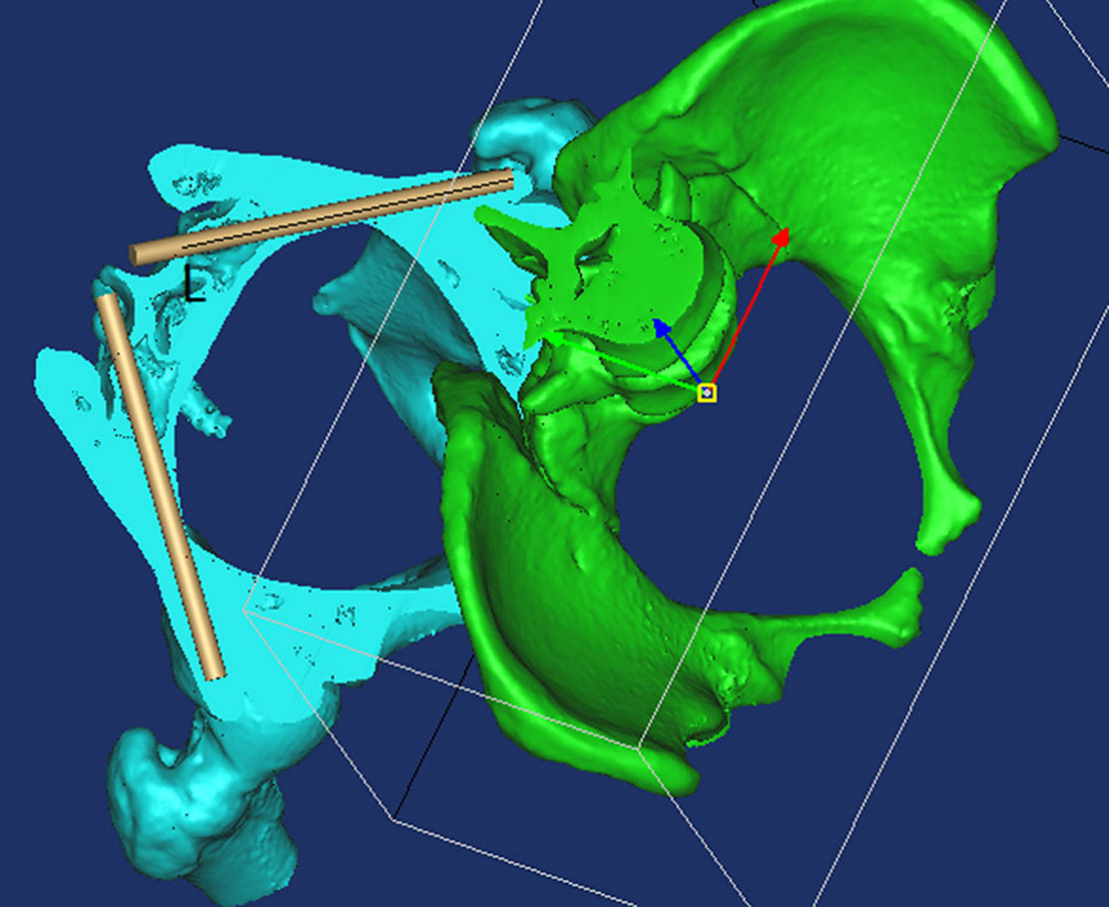
By using the “Cut,” “Split,” and “Reposition” functions in the Mimics software, the screw trajectory was clearly observed so that the screw trajectory could be adjusted conveniently. **L**, The length of the screw inside of bone.

The following three measurements were obtained for each imitated screw (Figs. 2 and 3): (1) **α**, the angle between the screw trajectory and the erect line from the anteroposterior view (Fig. 2A); (2) **β**, the angle between the screw trajectory and the erect line from the lateral view (Fig. 2B); and (3) **L**, the length of the screw inside of the bone (Fig. 3). Moreover, the possible application of percutaneous S3–4AI screw fixation for sacral fracture was noted and imitated. The measurements were performed by two surgeons (AMW and YXH), and the mean value was used for calculated, the Intraclass Correlation Coefficient (ICC) was calculated to assess how strongly the data from two surgeons resembled each other. The results were represented as “Mean ± Standard Deviation.”

## Results

Sacral AI screws could be successfully imitated by 3D digital imaging, the ICC between the data from two surgeons was 0.893, which is >0.800, means the data measured by two surgeons was strongly consistent to each other.

By observing the 3D images, we found that the ideal screw entry point was at the inner side of the transverse process of the sacrum from S1–S4 (Fig. 1) and that the screw could not be applied at S5. The S4AI screw trajectory could be obtained only in 19 of 45 patient images (42.2%); the feasibility rates of S1AI, S2AI, and S3AI were 100%, 100%, and 91.1% (41/45), respectively.

We found that from S1AI to S4AI, the α angle of maximum upward screw trajectory (Fig. 1A) was gradually increased from 54.5 ± 6.71° to 111.2 ± 4.90°, and the β angle of maximum upward screw trajectory (Fig. 1B) was gradually increased from 43.6 ± 5.44° to 110.7 ± 5.06°; while the α angle of maximum downward screw trajectory was gradually increased from 39.3 ± 3.10° to 107.3 ± 4.94°, the β angle was gradually increased from 30.1 ± 5.37°. And we found that the feasible region of the S1AI, S2AI, and S3AI screw trajectories was wide enough. The average adjustable α angles of S1AI, S2AI, and S3AI screw trajectories were 15.24°, 29.43°, and 19.61° in the left side and 15.03°, 29.47°, and 20.01° in the right side, respectively. In contrast, the average adjustable angle of S4AI screws was very small: only 4.05° in the left side and 3.58° in the right side. Therefore, it was difficult to insert S4AI screws due to the small region of feasibility. The measurement parameters of the S1–4AI screw trajectories are shown in Table 1.

**Table 1:** Parameters of S1–4AI screw trajectory measurements (Mean±standard deviation).

		Maximum upward screw trajectory			Maximum downward screw trajectory		
		α (°)	β (°)	L (mm)	α (°)	Β (°)	L (mm)
S1AI	Left	54.5 ± 6.71	43.7 ± 5.22	98.7 ± 5.29	39.3 ± 3.10	30.2 ± 5.64	102.1 ± 5.04
(N = 45)	Right	55.8 ± 5.95	43.6 ± 5.44	101.6 ± 5.48	40.8 ± 3.50	30.1 ± 5.37	103.3 ± 6.30
S2AI	Left	79.1 ± 6.33	66.9 ± 4.24	105.8 ± 5.12	49.7 ± 5.78	46.0 ± 3.91	94.7 ± 4.53
(N = 45)	Right	80.32 ± 4.66	67.7 ± 4.51	106.6 ± 5.16	50.85 ± 5.91	48.5 ± 4.16	95.2 ± 4.91
S3AI	Left	96.4 ± 6.57	94.2 ± 4.23	118.6 ± 6.58	76.8 ± 5.57	73.1 ± 3.38	96.4 ± 5.19
(N = 41)	Right	97.6 ± 5.89	95.4 ± 5.26	117.3 ± 6.07	77.5 ± 5.72	73.6 ± 4.21	95.9 ± 5.46
S4AI	Left	111.2 ± 4.90	110.0 ± 5.27	140.4 ± 4.75	107.1 ± 5.84	107.0 ± 5.59	128.2 ± 5.66
(N = 19)	Right	110.9 ± 4.56	110.7 ± 5.06	139.7 ± 3.60	107.3 ± 4.94	107.4 ± 5.23	126.8 ± 4.97

## Discussion

In this study, we determined the feasibility of S1AI screw fixation as well as S2AI, S3AI, and S4AI screw fixation and found that they were all inserted successfully in the adult population. By using the “Cut,” “Split,” and “Reposition” functions in the Mimics software, we could observe the screw trajectory clearly (Fig. 3). We found that the ideal screw entry point was not limited to a determined point but rather the inner fovea line of the transverse process of the sacrum from S1 to S4. In order to describe the trajectory of sacral AI screws in different segments, the entry point of the screw trajectory was chosen as the middle of two adjacent dorsal sacral foramina on the inner fovea line of the transverse process of the sacrum.

**Figure 4:**
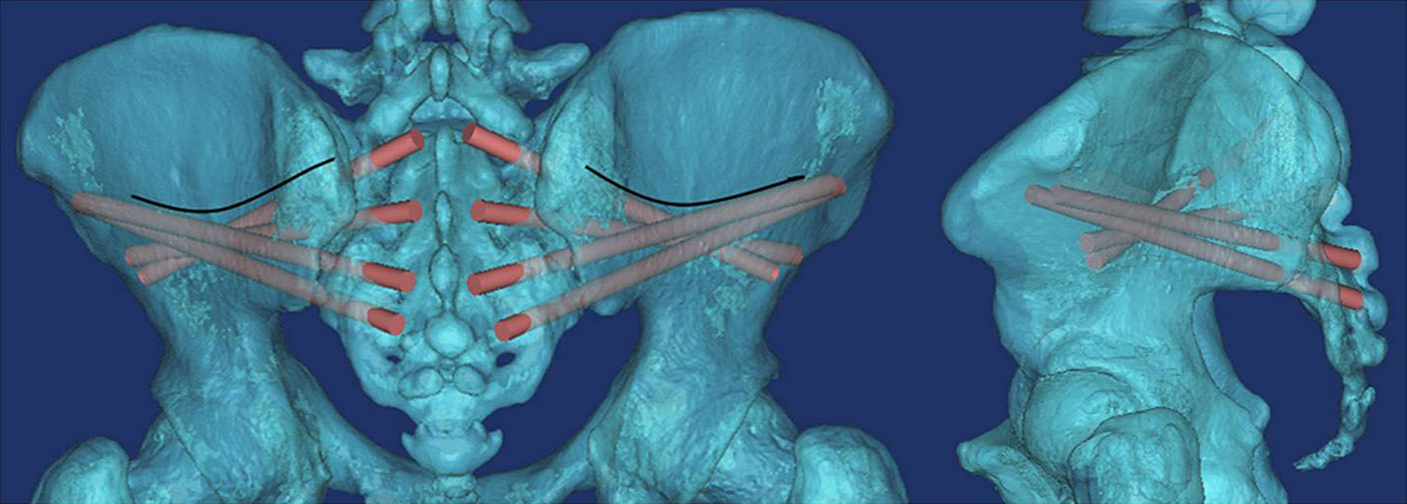
The region above the arc line is the iliac thin region. If the screw trajectory is adjusted to an upward angle that is too high, it will pierce the cortex of this region.

**Figure 5:**
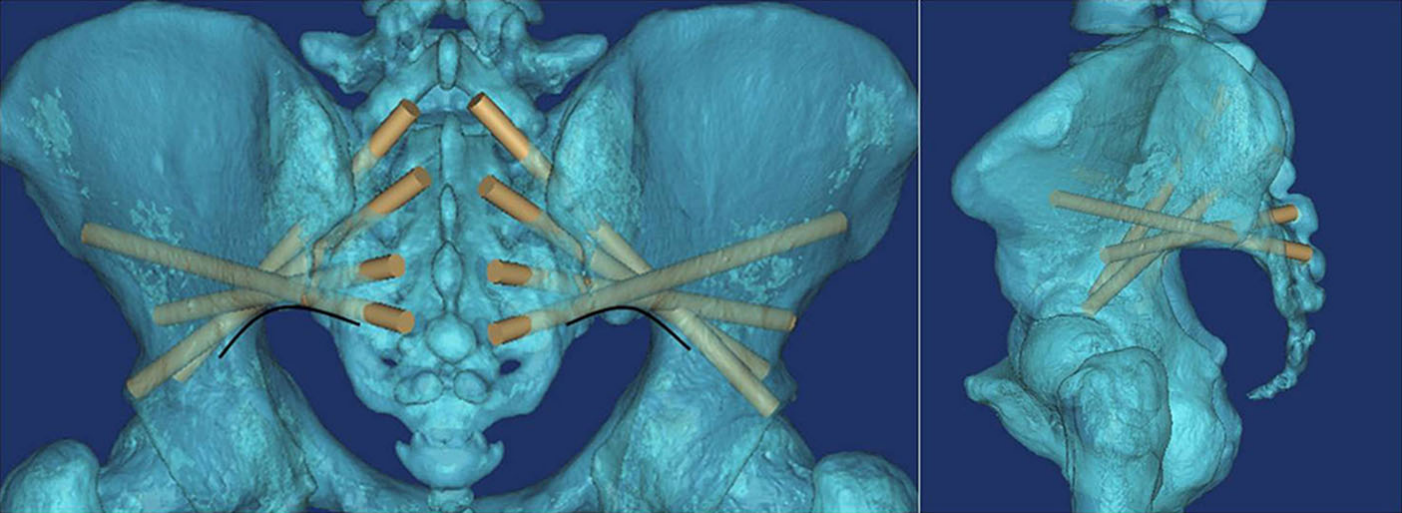
The arc line is the inferior border of the ilium. If the screw trajectory is adjusted to a downward angle that is too high, it will pierce the cortex of the inferior fovea of the ilium.

Every sacral AI screw trajectory in each segment had a maximum upward angle and a maximum downward angle. If the screw trajectory overstepped the feasible region between the maximum upward angle and the maximum downward angle, the screw will pierce the cortex of the iliac thin region (Fig. 4) or the cortex of the inferior fovea of the ilium (Fig. 5), respectively, and may injure peripheral soft tissue and vessels.

As shown in Table 1, the feasible region of the S1AI, S2AI, and S3AI screw trajectories was wide enough. The average adjustable α angles of S1AI, S2AI, and S3AI screw trajectories were 15.24°, 29.43°, and 19.61° in the left side and 15.03°, 29.47°, and 20.01° in the right side, respectively. In contrast, the average adjustable angle of S4AI screws was very small: only 4.05° in the left side and 3.58° in the right side. Therefore, it was difficult to insert S4AI screws due to the small region of feasibility.

## Value in clinical conditions

In this study, more than in previous S2AI screw fixation (Kwan et al., 2012; O’Brien et al., 2010; Zhu et al., 2013), we provide novel fixation of S1–AI, S3AI, and S4AI screws. All S1–4 AI screw trajectories were studied. Therefore, we provide an alternative fixation for surgeons, we believed that our alternative fixation could be performed on following clinical condition.

Firstly, the spine fixation may be extended to pelvic fixation, in S1, surgeons prefer to use an S1 pedicle screw. However, sometimes, the normal osseous structure is destroyed by a lytic lesion or trauma (Martin, Witham & Kebaish, 2011) or the application of a pedicle screw in S1 is impossible. For these situations, sacral AI screw fixation (S1–2AI screw fixation) is more suitable.

**Figure 6:**
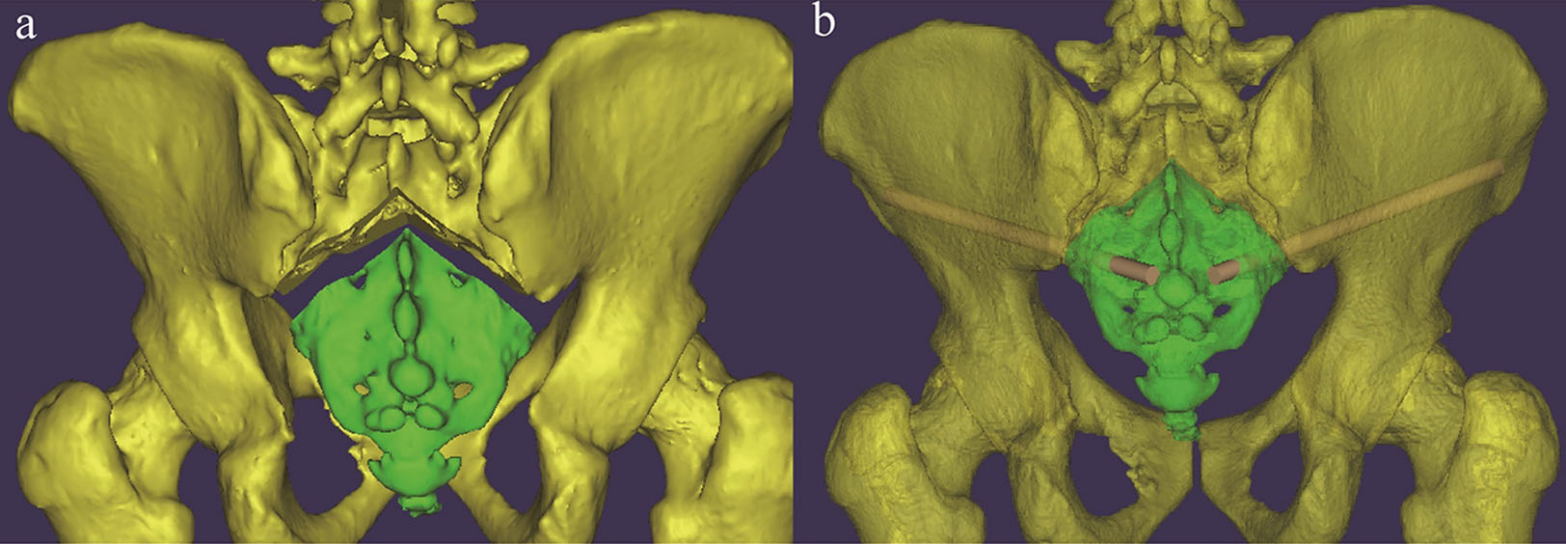
(A) Schematic diagram of a patient with a sacral fracture (Fracture was simulated in 3D images); (B) percutaneous S3–AI screw fixation was imitated after reduction.

Secondly, patients with a sacral fracture as shown in Fig. 6, which is uncommon in the clinic but does happen sometimes, percutaneous S3AI or S4AI screw fixation is suitable technique for them. Our results shown that S3–4AI screws could performed on part of the adult population; therefore, we suggested that a preoperative CT scan should be obtained and a simulated trajectory should be made to prove the feasibility of S3–4AI screws fixation on them.

Therefore, studying the feasibility of sacral AI screw fixation (including S3–4AI screws) has value, and the technique of sacral AI screw fixation has great potential value in clinical applications as it is theoretically better than traditional pedicle screw fixation and the sacropelvic fixation technique (O’Brien et al., 2013).

### Limitations

This is a 3D image study without clinical cases. In a 3D image study, we need some reference line to measure the angles of simulated trajectory. To determine the reference line, we adjusted the 3D image to simulate the patient is left-right symmetry stand position. Then, the erect line was made as the reference line; therefore, this line was subjective, and this is one of the limitation of our this study. In clinical cases, the position was also determined by surgeons subjectively; the angle will be a little different among different surgeons, but this will not influence the feasibility of S1–4 AI Screw fixation on patients. Meanwhile, because of individual variation, we strongly recommend that a preoperative CT scan is obtained and that a detailed preoperative plan is made before sacral AI fixation is applied. Moreover, 3D digital images were suitable to study the feasibility of new screw fixation and could help surgeons to explore the trajectory of screw fixation readily.

## Supplemental Information

10.7717/peerj.1587/supp-1 Supplemental Information 1 The raw data. The raw data from S1-AI to S40AI.

## Additional Information and Declarations

### Data Deposition

The following information was supplied regarding data availability: Raw data can be found in the Supplemental Information.
